# Another round, another chance: oocyte developmental competence and outcomes in second IVF attempts—the earlier, the better

**DOI:** 10.1007/s10815-026-03961-8

**Published:** 2026-07-03

**Authors:** Alessandro Ruffa, Danilo Cimadomo, Marilena Taggi, Federica Innocenti, Erika Pittana, Federica Battista, Gemma Fabozzi, Michele Morelli, Maurizio Guido, Laura Albricci, Giulia Fiorentino, Laura Rienzi, Filippo Maria Ubaldi, Alberto Vaiarelli

**Affiliations:** 1 Genera, Clinica Valle Giulia, IVIRMA Global Research Alliance, Rome, Italy; 2 Department of Biology and Biotechnology Lazzaro Spallanzani, University of Pavia, Pavia, Italy; 3 IVIRMA Italia, IVIRMA Global Research Alliance, Rome, Italy; 4 Reproductive Health Unit, IVIRMA Global Research Alliance, Rome, Italy; 5 Department of Biomedicine and Prevention, University of Rome Tor Vergata, Rome, Italy; 6 Department of Pharmacy and Health Sciences and Nutrition (DFSSN), University of Calabria, Rende, Italy; 7 Department of Biomolecular Sciences, University of Urbino, Urbino, Italy

**Keywords:** Time between consecutive cycles, Oocyte competence, IVF failure, Second IVF attempt, Multicycle approach

## Abstract

**Purpose:**

To evaluate whether the outcomes and characteristics of a first IVF/ICSI cycle are associated with embryological and clinical outcomes in a subsequent cycle performed within 2 years.

**Methods:**

This retrospective single-center observational study included 1190 couples undergoing two stimulated ICSI cycles with planned blastocyst culture between 2015 and 2020. Associations between first- and second-cycle outcomes were analyzed, including oocyte yield, blastocyst development, and live birth. The effect of the inter-cycle interval was assessed using multivariate models.

**Results:**

The mean maternal age was 38.7 years, and the mean AMH level was 1.7 ng/ml. Second cycles showed improved embryological outcomes, including higher numbers of retrieved cumulus–oocyte complexes (COCs), increased blastocyst yield, and higher blastocyst rate per COC. Overall, 50% of patients retrieved more COCs in the second cycle, and 87% of those with no COCs in the first cycle obtained ≥ 1 in the second. A longer inter-cycle interval was negatively associated with outcomes, reducing the likelihood of retrieving more COCs (OR 0.96 per month, 95% CI 0.93–0.98), obtaining more blastocysts (OR 0.95, 95% CI 0.93–0.98), and improving blastocyst rate (OR 0.96, 95% CI 0.94–0.99). First-cycle outcomes were not associated with live birth probability in the second cycle, which was instead associated with maternal age and the number of COCs previously retrieved.

**Conclusions:**

First-cycle outcomes should not discourage further attempts, as subsequent cycles may yield improved embryological results. A shorter interval between cycles represents a modifiable factor associated with better outcomes. These findings support early re-treatment and a multicycle counseling approach to optimize cumulative live birth rates, especially as maternal age and ovarian reserve remain the most important predictors of success even in second attempts.

**Supplementary Information:**

The online version contains supplementary material available at https://doi.org/10.1007/s10815-026-03961-8.

## Introduction

Success in assisted reproductive technology (ART) treatment relies on the optimization of both clinical strategies and biological processes. Ovarian stimulation protocols have progressively evolved to maximize follicular recruitment based on each patient’s ovarian reserve, and the personalization of the trigger method—whether with hCG, GnRH agonist, or dual trigger—enhances both oocyte yield and maturity, while ensuring patient safety. Equally pivotal are biological improvements: rigorous laboratory quality control, efficient workflow management, and the routine use of extended embryo culture to the blastocyst stage have collectively contributed to more accurate embryo selection and improved implantation rates.

However, even with the use of optimized stimulation protocols and advanced lab techniques in high-performance laboratories, the cumulative live birth rate (CLBR) following a concluded in vitro fertilization (IVF) cycle remains approximately 33% in the general population [1] and varies significantly according to maternal age and the number of oocytes retrieved [2–4]. This underscores a critical issue: only one in four women accurately estimated their own chances of IVF success, while the majority significantly overestimated their likelihood of achieving a live birth, resulting in a substantial gap between perceived and actual outcomes [5]. Therefore, evidence-based individualized prognostic counseling is essential, not only to align expectations with clinical reality, but also to support shared decision-making and mitigate the emotional impact of IVF treatment failure.

Treatment discontinuation after an initial failed IVF cycle is common and significantly reduces the overall effectiveness of fertility care [6]. A systematic review and meta-analysis found that early discontinuation before completing three IVF cycles is associated with a 15% reduction in pregnancy or live birth rates [7], although it might be prevented through healthcare professionals’ counseling and an interval between IVF cycles [8]. Rather than anticipating success from a single IVF cycle, a multicycle approach has been proposed to promote perseverance through multiple planned attempts, with the aim of maximizing CLBR per intention to treat [9–11]. Initial counseling should realistically address the potential for failure and anticipated negative outcomes of infertility treatment (poor ovarian response, absence of oocytes available for insemination, absence of blastocysts or euploid blastocysts available for transfer, or unsuccessful embryo transfer), while also emphasizing the potential benefits of additional IVF attempts to achieve a live birth. However, the extent to which the couple’s reproductive history (including prior failed IVF cycles, the absence of euploid blastocysts within embryo cohorts, previous implantation failures, and miscarriages) can predict future success remains an area of ongoing investigation [12–16].

Significant inter-cycle variability in ovarian stimulation responses and treatment outcomes has been observed both among different women with similar characteristics and across repeated IVF cycles in the same patient [15, 17]. This unpredictability, which current clinical factors still fail to fully explain or control, reinforces the rationale for pursuing multiple IVF attempts when clinically appropriate. Emerging evidence also suggests a “cohort effect”, whereby the blastocyst implantation potential may vary depending on the biological intrinsic characteristics of the oocyte cohort, beyond age, embryo ploidy status, and morphology [18].

In this context, identifying if and which patients might benefit most from continued treatment is crucial. This study aimed to evaluate how the outcome and features of a first failed IVF cycle affected embryological and clinical outcomes in a second attempt. This study may support individualized and well-informed patient counseling by offering more accurate estimates of success following an initial IVF failure.

## Materials and methods

### Study design and IVF procedures

A retrospective single-center observational study was conducted at a private IVF clinic in Rome (IVIRMA Global Research Alliance, Genera, Clinica Valle Giulia), including all stimulated ICSI cycles planned for blastocyst culture and followed by a second ovarian stimulation and oocyte retrieval within 2 years, between 2015 and 2020. Ovarian stimulation was performed through a flexible GnRH antagonist protocol combined with different types of gonadotropins (recombinant FSH (rFSH) with or without recombinant LH (rLH) or human menopausal gonadotropin (hMG)). Daily subcutaneous injections of GnRH antagonist, either cetrorelix or ganirelix (Cetrotide® 0.25 mg, Merck KGaA, Germany, or Orgalutran® 0.25 mg, Organon, USA), were added when the follicular diameter exceeded 13 mm until the day of trigger. FSH starting dose (150 IU, 225 IU, or 300 IU) was decided according to ovarian reserve and previous ovarian response (expected high, normal, and poor responders, respectively). LH activity (via hMG or rLH) was added based on physicians’ decision. Transvaginal ultrasound monitoring was conducted to assess follicular growth. Once at least three follicles reached a diameter ≥ 18 mm, final follicular maturation was triggered through either hCG 10,000 IU (Gonasi®, IBSA, Switzerland) or GnRH-agonist with 0.3 mg of triptorelin acetate (Decapeptyl®, IPSEN, France). The latter was used in case of expected hyper-response. Oocyte retrieval was performed 35–36 h after trigger. Every follicle above 10 mm was bilaterally aspirated under ultrasound guidance. Only ICSI on all metaphase-II (MII) oocytes and extended embryo culture to the blastocyst stage were conducted, as described previously [19–21].

In the case of the DuoStim protocol, a second consecutive ovarian stimulation was initiated 5 days after the first oocyte retrieval, using the same stimulation regimen [22]. Both the first and the second consecutive stimulations within a DuoStim cycle were considered separate cycles for statistical analysis.

In the case of preimplantation genetic testing for aneuploidies (PGT-A), trophectoderm biopsy was performed on fully expanded blastocysts without day-3 zona pellucida-drilling. The simultaneous zona opening and trophectoderm biopsy protocol was adopted, as described previously [21, 23]. PGT-A was suggested in case of advanced maternal age (≥ 35 years), repeated implantation failure and/or recurrent pregnancy loss, or patients’ request [24], and performed through comprehensive chromosome testing to identify whole chromosome uniform aneuploidies at an external genetic lab [25–27].

There were no significant changes in the laboratory’s main standard operating procedures during the study period.

Both fresh and vitrified-warmed untested/euploid embryo transfers were included in the study.

Endometrial preparation protocol was chosen according to patients’ characteristics and physicians’ judgement. Vitrified-warmed ETs were performed with either a modified natural cycle (mNC) or after hormone replacement therapy (HRT). In case of HRT, 2 mg oral estradiol valerate (Progynova®, Bayer, Germany) was administered three times a day from the second day of the period. When the endometrial thickness reached at least 7 mm and the aspect was trilaminar, vaginal micronized progesterone 800 mg/day was administered (Progeffik®, Effik, Italy) from day 0 to support the luteal phase. ET was scheduled after 6 days of micronized progesterone. The HRT was administered until the 10th week of gestation in case of pregnancy. In case of a mNC, a single dose of 10,000 IU hCG was administered when the leading follicle was > 17 mm and the endometrium measured ≥ 7 mm with a trilaminar aspect, along with 600 mg/day of micronized vaginal progesterone (Progeffik®) for luteal phase support starting 36–40 h post-hCG administration (day 0). ET was scheduled 7 days after hCG injection. All blastocysts were transferred about 2 h after warming.

We examined the association between characteristics of the first cycle and the main outcomes of the second attempt, namely the likelihood of retrieving more cumulus-oocyte complexes (COCs) and obtaining more blastocysts, the oocyte developmental competence defined as blastocyst rate per COCs, and the CLBR per cycle defined as the chance of achieving at least one live birth among concluded cycles (i.e., live birth achieved or all embryos transferred without achieving a live birth) [28]. Specifically, the characteristics of the first attempt which were investigated for their association are as follows: maternal age, cause, type and duration of infertility, basal hormonal values, severe male factor, embryological and clinical outcomes achieved, months between consecutive attempts, adoption of a DuoStim protocol, differences in LH activity adoption and FSH starting dose, and trigger methods.

Ethics committee approval was obtained for the retrospective analysis of pseudonymized data to investigate the efficacy and efficiency of IVF protocols and strategies.

### Statistical analysis

*T*-tests/ANOVA or Mann–Whitney *U*/Kruskal–Wallis tests were performed after controlling the Gaussian distribution of continuous data, and Fisher’s exact/chi-squared tests for categorical data. Pearson’s correlation coefficients were calculated for all the results after the first and second attempts. Univariate/multivariate linear/logistic regressions were performed to confirm associations. SPSS (IBM, USA) was used for all statistics.

## Results

### Patient and ovarian stimulation characteristics

A total of 1190 couples were included in the study. Baseline patients’ characteristics are summarized in Table 1.

**Table 1:** Couples’ characteristics

Couples’ characteristics (*N* = 1190)	
Main cause of female infertility, *n*, %	
Idiopathic	*N*=79, 6.6%
Endometriosis	*N*=70, 5.9%
Endocrine-Ovulatory	*N*=15, 1.3%
Tubal	*N*=66, 5.5%
Advanced maternal age	*N*=916, 77%
Reduced ovarian reserve	*N*=44, 3.7%
Severe Male Factor Infertility, *n*, %	
Absent	*N*=966, 81.2%
Present	*N*=224, 18.8%
Maternal age, mean ± SD	38.7 ± 3.8 years
AMH, mean ± SD	1.7 ± 1.5 ng/ml
FSH, mean ± SD	10.4 ± 3.9 IU/ml
estradiol, mean ± SD	44.7 ± 17.6 pg/ml
LH, mean ± SD	6.0 ± 2.9 IU/ml
Previous conceptions, *n*, %	
No	852, 71.6%
Yes	338, 28.4%
Duration of infertility, mean ± SD	2.9 ± 1.8 years

*AMH* Anti-müllerian hormone, *FSH* Follicle-stimulating hormone, *LH* Luteinizing hormone.

The mean maternal age was 38.7 years, and the mean AMH level was 1.7 ng/ml. A severe male factor infertility was identified in 18.8% of cases.

The average interval between the first and second attempt was 3.1 months, as illustrated in Supplemental Fig. 1. A total of 336 patients (28%) underwent a second attempt within a DuoStim protocol, and in 699 of the 796 first attempts with ≥ 1 blastocyst, PGT-A was adopted (87.8%).

Regarding ovarian stimulation characteristics, the duration of stimulation was longer in 41% of cases, equal in 27%, and shorter in 32% (Supplemental Fig. 2A). Overall, 66% of patients received the same FSH starting dose in both cycles, while in the remaining cases, the dosage was adjusted according to the ovarian response observed during the first stimulation. An increase in the FSH starting dose during the second cycle was observed in 18% of cases. Overall, a fixed dose of 300 IU was administered in most patients (52%) (Supplemental Fig. 2B). At last, 65% of the patients were administered the same type of gonadotropin (rFSH or hMG/rFSH-rLH) (Supplemental Fig. 2C) and the same ovulation trigger in both stimulation cycles (Supplemental Fig. 2D).

### Comparison between the first and second IVF attempts

In general, a significant improvement in all main outcomes was observed in second cycles, including larger cohorts of COCs retrieved (+ 0.7, 95% CI + 0.5 to + 1, *p* < 0.001), more blastocysts obtained (+ 0.5, 95% + 0.3 to + 0.6, *p* < 0.001), and higher blastocyst rate per COCs (+ 3.3%, 95% CI + 1.8% to + 4.8%, *p* < 0.001; Table 2).

**Table 2:** Comparison of the embryological outcomes between the first and second IVF attempts

	Attempt 1 Mean ± SD	Attempt 2 Mean ± SD	Person’s correlation, *p*-value	Delta 2nd vs 1 st attempt, 95% CI, *p*-value
COCs retrieved	7.8 ± 5.8	8.6 ± 6.3	0.74, *p* < 0.001	+ 0.7, 95% CI + 0.5 to + 1, *p* < 0.001
Blastocysts obtained	1.5 ± 1.6	2.0 ± 2.0	0.40, *p* < 0.001	+ 0.5, 95% + 0.3 to + 0.6, *p* < 0.001
Blastocyst rate per COCs in 1101 couples with COCs obtained at both attempts	17.9% ± 20.1%	21.2% ± 20.9%	0.21, *p* < 0.001	+ 3.3%, 95% CI + 1.8% to + 4.8%, *p* < 0.001
Maturation rate per COCs in 1101 couples with COCs obtained at both attempts	67.2% ± 23.7%	69.5% ± 22.4%	0.17, *p* < 0.001	+ 2.3%, 95% CI + 0.5% to + 4.1%, *p* = 0.010
Fertilization rate per MII in 1057 couples with MIIs obtained at both attempts	67.5% ± 27.0%	70.0% ± 24.7%	0.15, *p* < 0.001	+ 2.5%, 95% CI + 0.5% to + 4.5%, *p* = 0.015
Blastocyst rate per 2PN in 980 couples with 2PNs obtained at both attempts	39.7% ± 32.6%	42.9% ± 31.1%	0.22, *p* < 0.001	+ 3.2%, 95% CI + 0.7% to + 5.7%, *p* = 0.012

*COCs* Cumulus-oocyte complexes, *MII* Metaphase II, *2PN* Two pronuclei, *CI* Confidence interval.

The correlation between second cycles and improved outcomes was strongest for the number of COCs retrieved (Pearson’s correlation coefficient, 0.74), while weaker associations were found for the other embryological outcomes evaluated, including number of blastocysts obtained and blastulation rate (Table 2). A similar trend was also observed in intermediate outcomes, such as maturation and fertilization rates (Table 2).

**Fig. 1:**
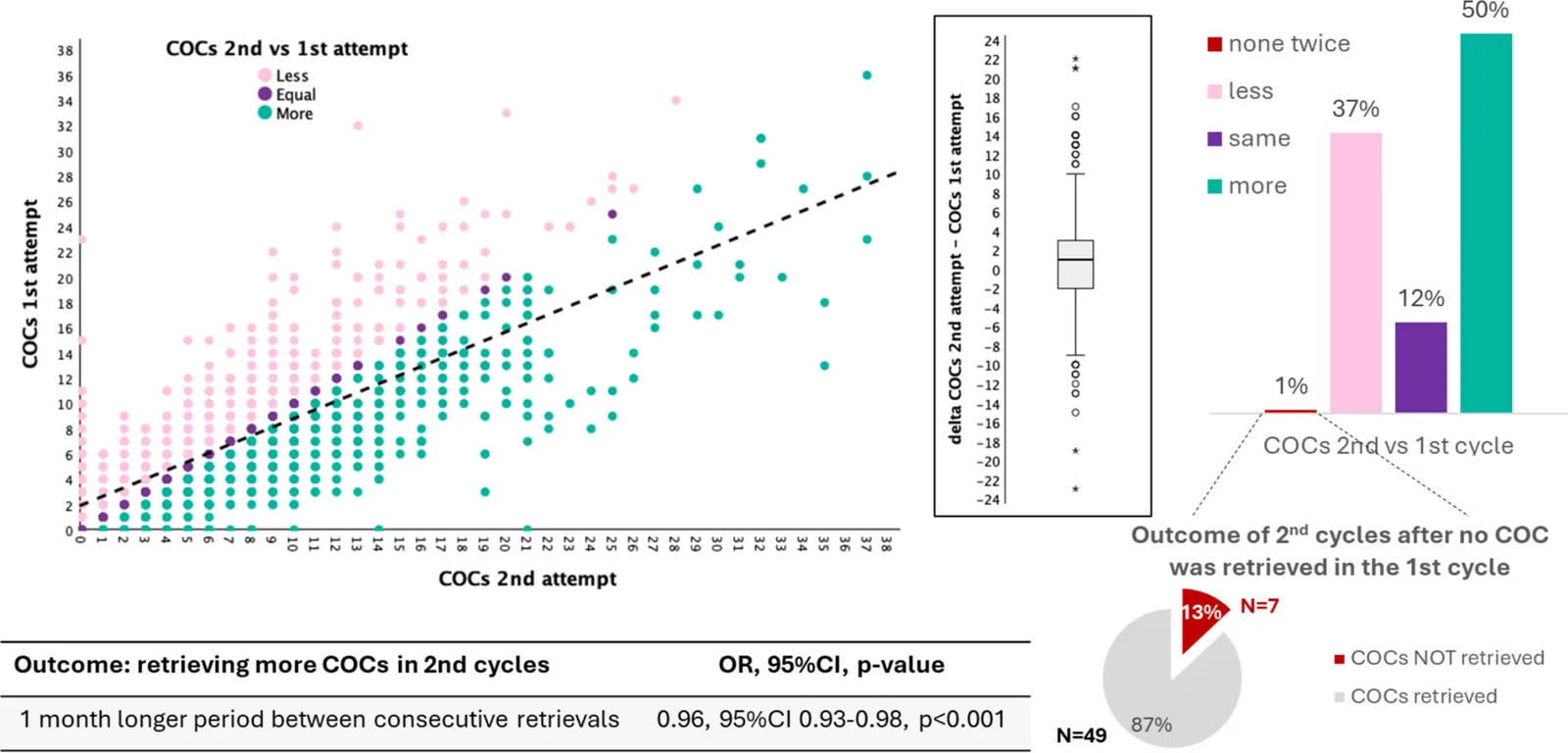
Differences in retrieved cumulus–oocyte complexes (COCs) between the first and second IVF attempts. **A** Scatter plot illustrating the number of COCs retrieved during the first and second attempts. **B** Box plot showing the distribution of differences (delta) in the number of COCs between the two attempts. **C** Histogram depicting the prevalence of second-cycle outcomes compared with the first, including repeated absence of oocytes retrieved, a decrease, no change, or an increase in the number of COCs. **D** Univariate logistic regression analysis showing the association between a 1-month longer interval between consecutive retrievals and the likelihood of retrieving a higher number of COCs in the second cycle. COCs, cumulus-oocyte complexes; OR, odds ratio; CI, confidence interval

In the second oocyte retrieval attempt, 12% of patients obtained the same number of COCs, while 50% retrieved a higher number. Oocyte retrieval failure in both consecutive cycles occurred in only 1% of all patients. Notably, 87% of those with no oocytes yielded from the first cycle (*N* = 49) obtained COCs in the second attempt. A shorter interval between retrievals was significantly associated with a higher likelihood of retrieving more COCs. Specifically, each additional month between cycles was linked to a 4% relative reduction in this chance (OR 0.96, 95% CI 0.93–0.98, *p* < 0.001) (Fig. 1). The change in FSH starting dose between the two attempts showed no association with this outcome (decreased vs same dose aOR 0.79, 95% CI 0.58–1.08, *p* = 0.144; increased vs same dose aOR 1.19, 95% CI 0.89–1.62, *p* = 0.283). Similarly, the removal or addition of LH activity was not associated with an increase in the COCs retrieved (removed aOR 1.06, 95% CI 0.77–1.47, *p* = 0.722; added aOR 1.24, 95% CI 0.93–1.67, *p* = 0.149). Neither the adoption of a second stimulation in the same ovarian cycle (DuoStim) was associated with this outcome in the multivariate regression (aOR 1.07, 95% CI 0.79–1.43, *p* = 0.681).

**Fig. 2:**
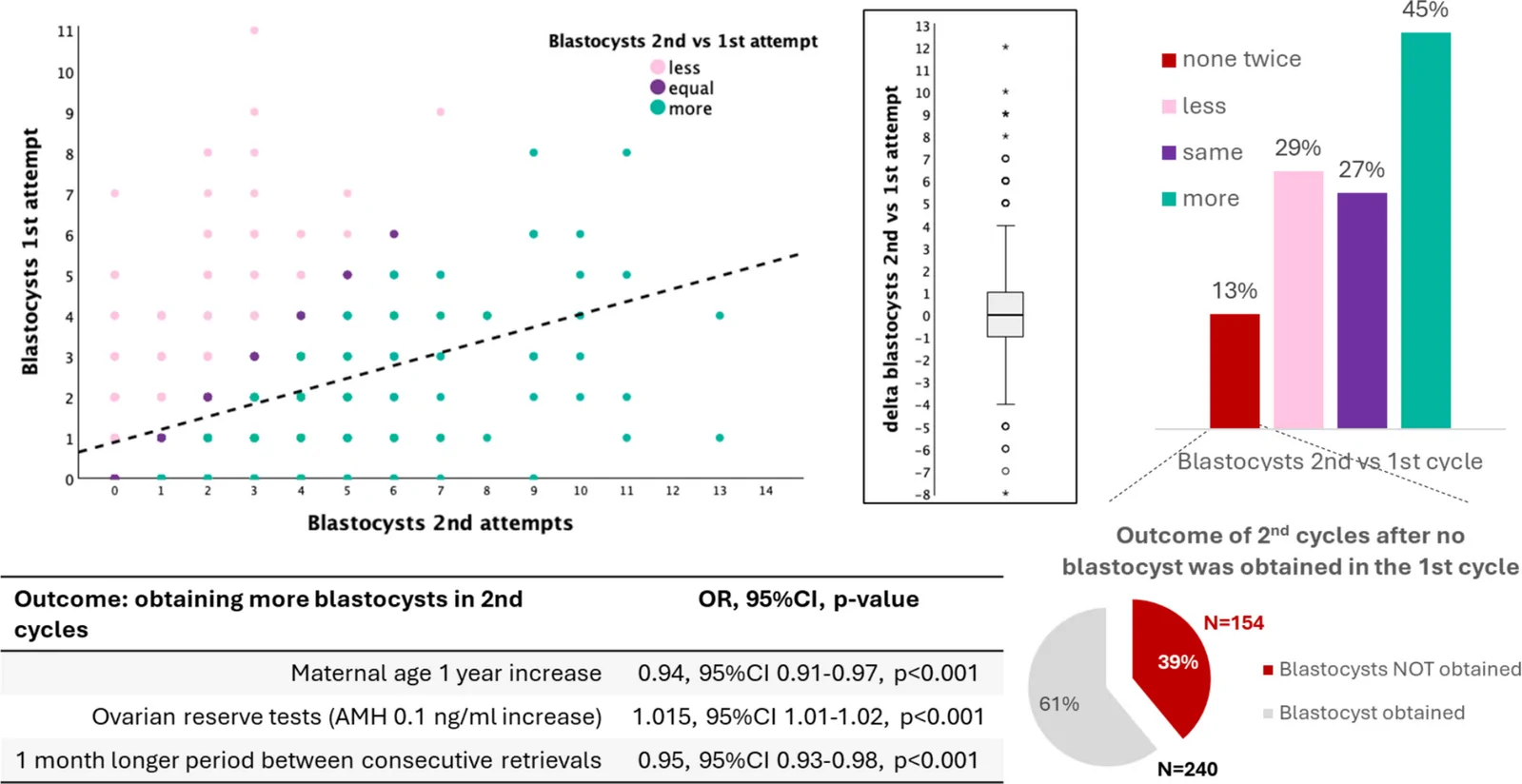
Differences in obtained blastocysts between the first and second IVF attempts. **A** Scatter plot illustrating the number of blastocysts obtained during the first and second attempts. **B** Box plot showing the distribution of differences (delta) in the number of blastocysts between the two attempts. **C** Histogram depicting the prevalence of second-cycle outcomes compared with the first, including repeated absence of blastocysts obtained, a decrease, no change, or an increase in the number of blastocysts. **D** Multivariate logistic regression analysis showing the association between 1-month longer intervals between consecutive retrievals, AMH, and 1-year increase in maternal age and the likelihood of obtaining a higher number of blastocysts in the second cycle. AMH, anti-müllerian hormone; OR, odds ratio; CI, confidence interval

Among patients who failed to obtain a blastocyst in their first attempt, 39% experienced the same outcome in the second attempt, representing 13% of the overall cohort. Conversely, 27% obtained the same number of blastocysts as in the first cycle, while 45% achieved a higher number. Maternal age (per 1-year increase) was negatively associated with blastocyst yield (OR 0.94, 95% CI 0.91–0.97, *p* < 0.001), whereas AMH levels (per one-unit increase) were positively associated (OR 1.02, 95% CI 1.01–1.02, *p* < 0.001). In contrast, a longer interval between attempts (per 1-month increase) was associated with a reduced likelihood of obtaining a higher blastocyst yield (OR 0.95, 95% CI 0.93–0.98, *p* < 0.001). Specifically, each 1-year increase in maternal age was associated with a 6% relative decrease in the likelihood of a higher number of blastocysts obtained, while each additional month between cycles was linked to a 5% lower chance of achieving more blastocysts (Fig. 2). The change in FSH starting dose between the two attempts showed no association with this outcome (decreased vs same dose aOR 1.08, 95% CI 0.79–1.48, *p* = 0.618; increased vs same dose aOR 0.99, 95% CI 0.73–1.37, *p* = 0.987). Similarly, the removal or addition of LH activity was not associated with an increase in the blastocysts obtained (removed aOR 0.91, 95% CI 0.66–1.27, *p* = 0.594; added aOR 1.10, 95% CI 0.82–1.49, *p* = 0.529). Neither the adoption of a second stimulation in the same ovarian cycle (DuoStim) was associated with this outcome in the multivariate regression (aOR 0.76, 95% CI 0.56–1.03, *p* = 0.081).

**Fig. 3:**
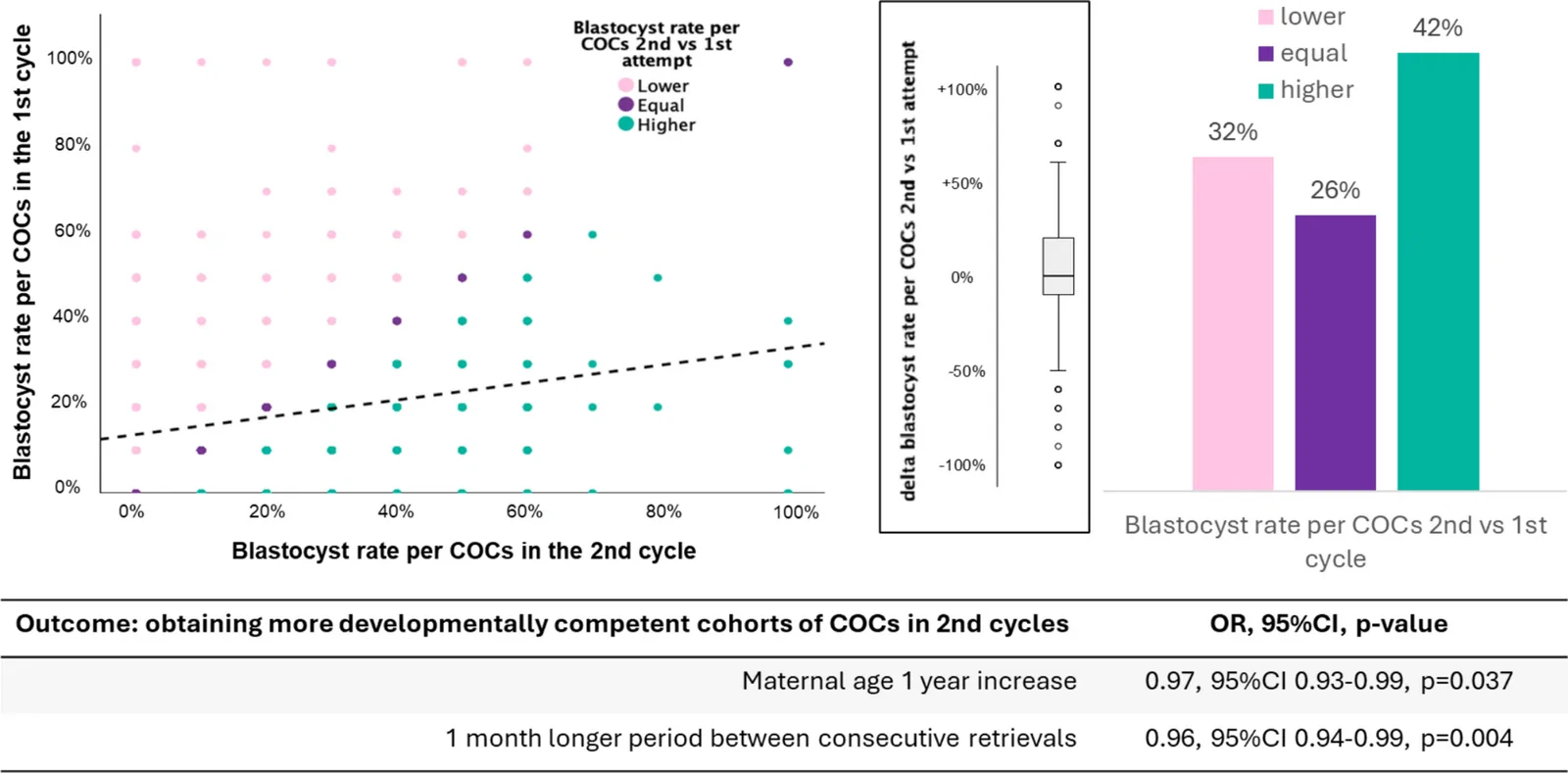
Differences in blastocyst rate per cumulus-oocyte complexes (COCs) between the first and second IVF attempts. **A** Scatter plot illustrating the blastocyst rate per COCs during the first and second attempts. **B** Box plot showing the distribution of differences (delta) in the blastocyst rate per COCs between the two attempts. **C** Histogram depicting the prevalence of second-cycle outcomes compared with the first, including a decrease, no change, or an increase in the blastocyst rate per COCs. **D** Multivariate logistic regression analysis showing the association between a 1-month longer interval between consecutive retrievals and a 1-year increase in maternal age and the likelihood of obtaining a higher number of blastocysts per cohort of COCs in the second cycle. COCs, cumulus-oocyte complexes; OR, odds ratio; CI, confidence interval

Oocyte developmental competence, defined as the blastocyst rate per retrieved COCs, remained unchanged in 26% of patients and improved in 42% during the second attempt. Maternal age (OR 0.97, 95% CI 0.93–0.99, *p* = 0.04) and the time between attempts (OR 0.96, 95% CI 0.94–0.99, *p* = 0.004) were associated with a lower likelihood of improvement. Specifically, each 1-year increase in maternal age was associated with a 3% reduction in the chance of obtaining a higher blastulation rate, while each additional month between cycles was linked to a 4% decrease (Fig. 3). The change in FSH starting dose between the two attempts showed no association with this outcome (decreased vs same dose aOR 1.20, 95% CI 0.86–1.66, *p* = 0.279; increased vs same dose aOR 1.02, 95% CI 0.74–1.43, *p* = 0.890). Similarly, the removal or addition of LH activity was not associated with an increase in oocyte competence (removed aOR 0.99, 95% CI 0.70–1.42, *p* = 0.968; added aOR 1.07, 95% CI 0.79–1.46, *p* = 0.652). Neither the adoption of a second stimulation in the same ovarian cycle (DuoStim) was associated with this outcome in the multivariate regression (aOR 0.76, 95% CI 0.55–1.04, *p* = 0.090).

**Fig. 4:**
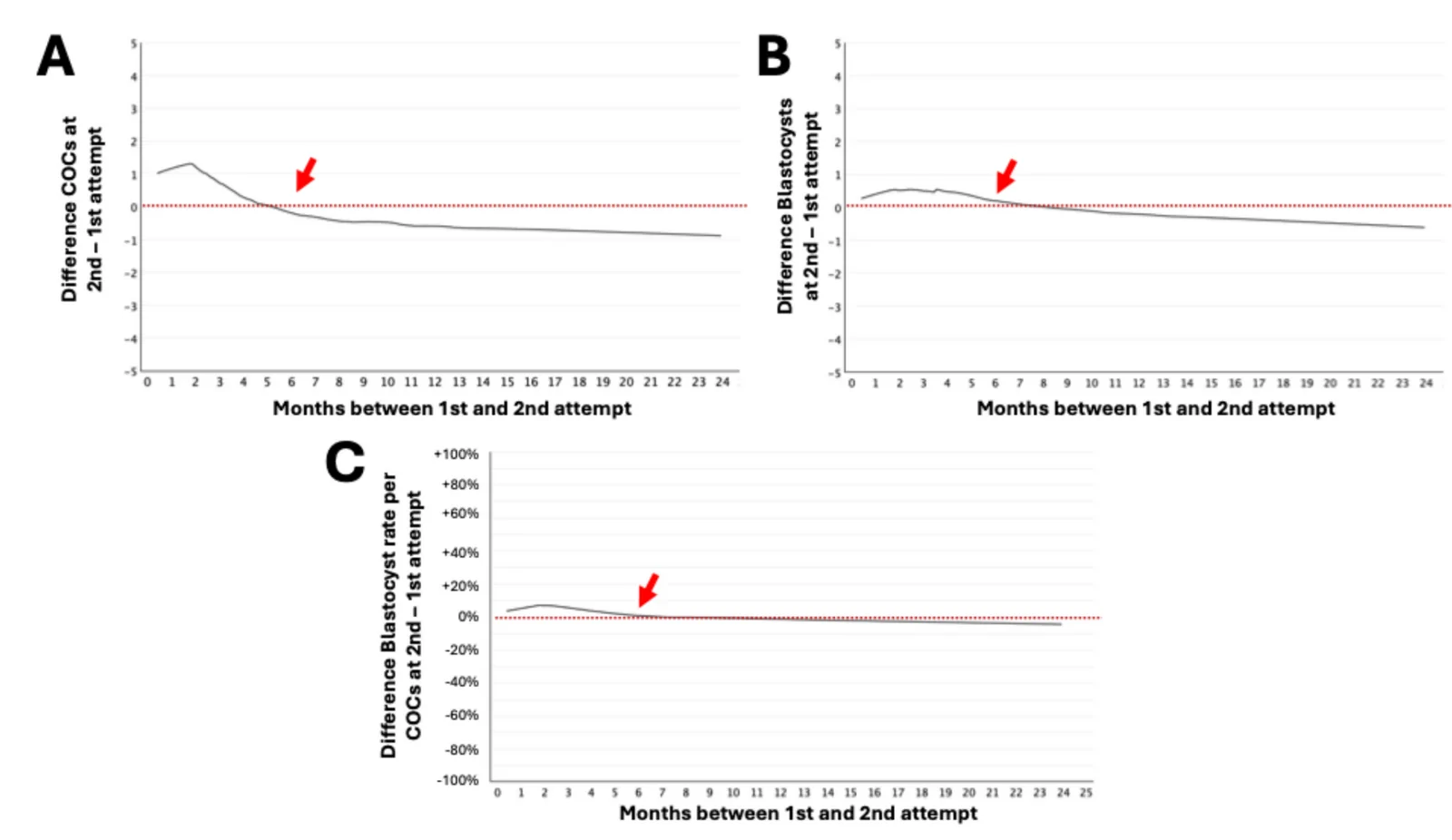
Effect of the inter-cycle interval on differences in cumulus–oocyte complexes (COCs) and blastocyst yield and blastulation rate. A higher number of cumulus–oocyte complexes (COCs) and slightly more blastocysts are often obtained when the second attempt is performed within 6 months of the first. The black lines represent the locally estimated scatterplot smoothing (LOESS) curves and illustrate the association between the interval (months) between consecutive attempts and the difference in **A** COCs, **B** blastocysts, and **C** blastocyst rate per COCs between consecutive attempts. The dotted red line defines the absence of difference between the two attempts. The red arrowheads identify the sixth month after the first retrieval, namely when all differences in the outcomes begin to plateau or turn negative. COCs, cumulus-oocyte complexes

The overall effect of the inter-cycle interval, defined as the number of months between the first and second attempts, on differences in oocyte and blastocyst yield, and blastulation rate is illustrated in Fig. 4.

Following regression analysis, no significant association was found between severe male factor infertility, including azoospermia, and the primary outcomes of the study.

An IVF cycle was considered concluded either when a live birth was achieved or when all available embryos had been transferred. Among the 1190 couples included, 1134 completed both cycles. Of the 1089 patients who did not achieve a live birth after the first attempt (with or without DuoStim), 253 obtained at least one live birth in the second attempt, resulting in a CLBR per second attempt of 23.2%. Among the 45 patients with a live birth from the first attempt (CLBR per first attempts: 24.4%), 11 achieved an additional live birth in the second cycle. The likelihood of achieving a live birth in the second cycle was not significantly influenced by whether a live birth was achieved in the first cycle, even after adjusting for AMH (OR 0.82, 95% CI 0.41–1.63, *p* = 0.568). Maternal age (OR 0.89, 95% CI 0.86–0.92, *p* < 0.001) and the number of COCs retrieved during the first attempt (OR 1.06, 95% CI 1.04–1.09, *p* < 0.001) were the only factors significantly associated with CLBR. Specifically, each 1-year increase in maternal age was associated with an 11% decrease in the chance of achieving a live birth in the second cycle (Table 3). The change in FSH starting dose between the two attempts showed no association with this outcome (decreased vs same dose aOR 0.81, 95% CI 0.55–1.21, *p* = 0.309; increased vs same dose aOR 1.15, 95% CI 0.79–1.65, *p* = 0.468). Similarly, the removal or addition of LH activity was not associated with an increase in the chance of achieving a live birth at the second attempt (removed aOR 1.10, 95% CI 0.74–1.65, *p* = 0.641; added aOR 0.79, 95% CI 0.54–1.14, *p* = 0.202). Neither the adoption of a second stimulation in the same ovarian cycle (DuoStim) was associated with this outcome in the multivariate regression (aOR 1.02, 95% CI 0.71–1.47, *p* = 0.915).

**Table 3:** Multivariate logistic regressions analysis showing the association between a 1 year increase in maternal age and the number of COCs retrieved at first attempt and the chance of achieving a live birth in the second cycle

Outcome: chance of a live birth (LB) in a second cycle	OR, 95% CI, *p*-value
Maternal age 1 year increase	0.89, 95% CI 0.86–0.92, *p* < 0.001
Number of COCs retrieved at first attempt	1.06, 95% CI 1.04–1.09, *p* < 0.001
LBs achieved at 1 st attempt (yes vs no)	0.82, 95% CI 0.41–1.63, *p* = 0.568

*COCs* Cumulus-oocyte complexes, *LBs* Live births, *OR* Odds ratio, *CI* Confidence interval.

**Fig. 5:**
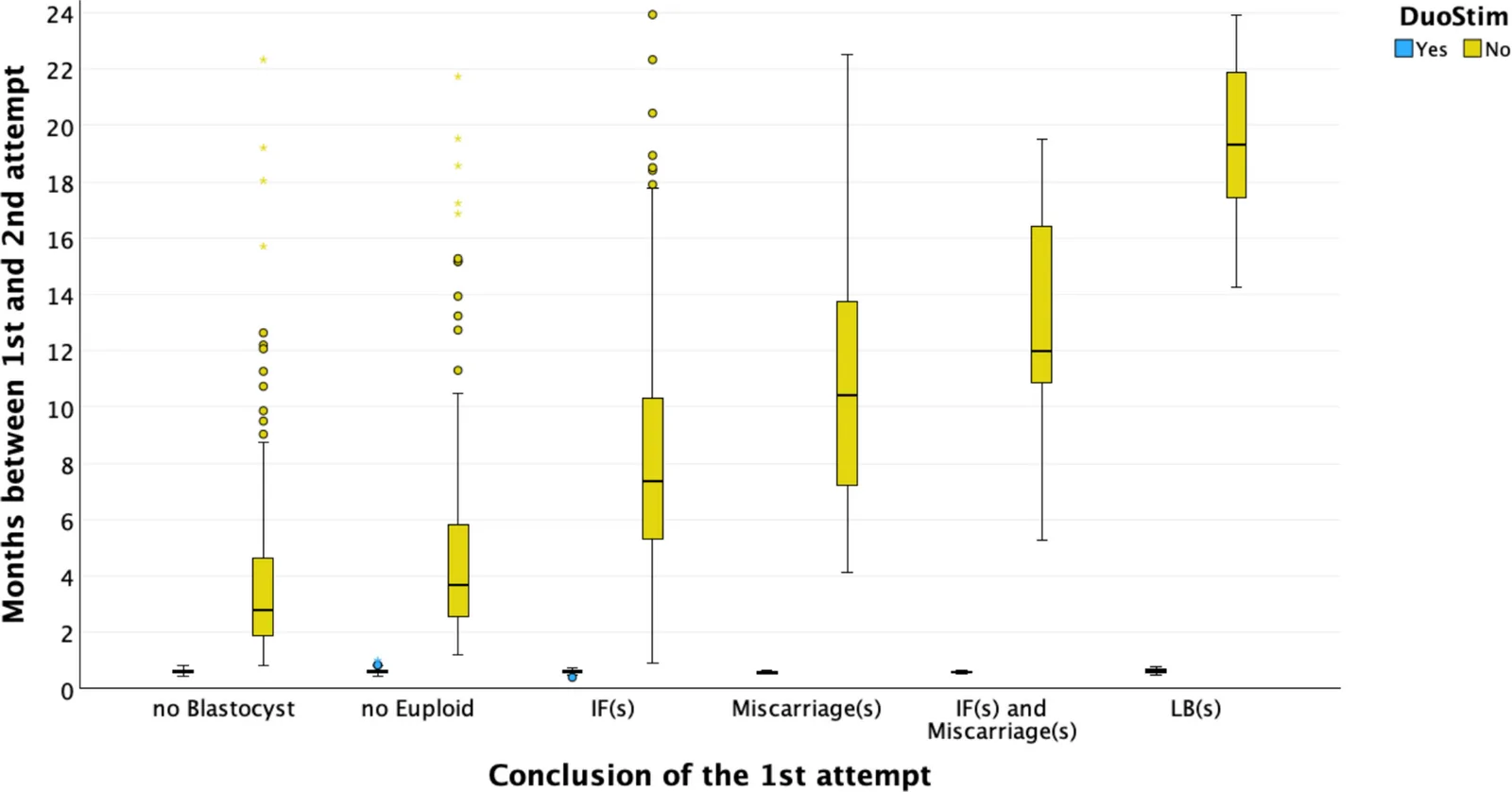
Interval between consecutive oocyte retrievals, stratified by the reason for first-cycle conclusion and by the adoption of a multicycle approach through DuoStim. This reports the median value in months for each group along with the first and third quartiles. Data are shown for all patients except for couples who underwent DuoStim and still have transferable embryos without a live birth being achieved from the first oocyte retrieval (*N* = 12 out of 1190). ET, embryo transfer; IF, implantation failure; LB, live birth

The time between two consecutive IVF cycles varied considerably depending on the outcome of the first cycle, with longer intervals observed following miscarriage or implantation failure, as shown in Fig. 5.

The use of the DuoStim protocol resulted in considerable time saving regardless of the outcome of the first attempt, including cases in which a live birth was achieved. Interestingly, in cycles without DuoStim, the prevention of aneuploid embryo transfers via PGT-A resulted in a time to the second attempt of approximately 3.5 months, which was significantly shorter than the intervals observed for couples after implantation failure and/or miscarriage.

## Discussion

Managing failure represents a major challenge in ART, as it poses both clinical and emotional burdens for couples. The first IVF attempt often carries the highest expectations, and its failure can lead to frustration and, ultimately, discontinuation of treatment before achieving a live birth. Reported discontinuation rates range from 37 to 50% [6, 29], with nearly one in four patients already abandoning treatment after a first failed attempt [29]. This phenomenon is only partially imputable to economic factors. Indeed, even in healthcare systems where infertility treatments are fully covered, high rates of early discontinuation have been reported following unsuccessful cycles [30].

Several modifiable factors, including the adequacy of counseling and the timing of subsequent IVF cycles, emerge as key determinants of patient compliance. These may be effectively addressed through targeted clinical and psychological interventions aimed at preventing early discontinuation [7, 8]. In this context, understanding the likelihood of achieving favorable outcomes after a first failed IVF attempt is essential for guiding clinical decisions and counseling.

Our analysis indicates that failure during the first IVF attempt, whether due to cycle cancellation because of monofollicular growth (2.8% of 1190 patients), absence of retrieved oocytes (1.9%), or lack of blastocyst development (33%), does not necessarily predict a similar outcome in subsequent cycles. Only 1% of patients failed to obtain oocytes twice, while 87% of those who retrieved none during the first attempt obtained ≥ 1 oocyte in a second cycle. This is particularly relevant, as data on cancelled IVF cycles are scarce and the prevalence of patients collecting no oocytes at pick-up is approximately 5%, mostly among poor responders [31], with limited evidence in the general IVF population [32].

Half of the patients in our study retrieved more COCs in their second attempt, consistent with previous reports [12, 33]. Other studies stratified by ovarian reserve or age found significant increases in oocyte yield between cycles only among women with normal ovarian reserve [33] or those under 38 years old [12]. Such improvement has been attributed to stimulation-related factors, particularly a higher total gonadotropin dose in the second cycle [12, 33]. However, in our study, neither the use of LH activity nor FSH starting dose, stimulation duration, nor the trigger method were associated with this outcome, which appeared to be influenced mainly by the interval between retrievals.

A substantial degree of unexplained variability in ovarian response persists even among women with similar characteristics or across repeated cycles in the same individual [17]. This heterogeneity cannot yet be predicted or mitigated based on current knowledge and actionable factors. As an example, the practitioner performing the pick-up has been reported to account for only about 19% of oocyte yield variation between consecutive cycles [17].

In this study, the increased number of COCs retrieved during a second cycle performed shortly within 5 months from the first may reflect the combined action of several mechanisms. First, a short- to medium-term effect of the first cycle can be hypothesized. During the first stimulation, growing antral follicles secrete factors that diffuse and modulate the ovarian endocrine and paracrine environment, thus promoting the transition of secondary follicles to the gonadotropin-responsive early-antral stage. Specifically, active growing antral follicles secrete TGF-β family members and KIT ligand [34], which diffuse locally to support secondary-follicle survival and growth. Following oocyte retrieval, removal of dominant follicles induces an abrupt decline in estradiol and inhibin B [35–37] and an increase in IGF bioavailability, enabling IGF and FSH to act synergistically to promote granulosa-cell proliferation and upregulate FSHR expression [38, 39]. Because secondary follicles need about 2–3 months to reach the gonadotropin-responsive early-antral stage [40], they become recruitable for a second stimulation cycle, a time interval which is consistent with the observed median interval of 3.1 months between the consecutive oocyte retrievals. Interestingly, this positive effect seems to vanish 6 months after the first retrieval, if not being reversed probably because of the counteracting effect of increasing maternal age. In the context of a DuoStim protocol, a potential GnRH agonist may trigger a “flare-up” effect that induces down-regulation of follicular AMH expression and/or improved synchronization of follicles within the second anovulatory wave, thereby enhancing their responsiveness to stimulation (35–37). Nevertheless, the adoption of a second stimulation in the same ovarian cycle was not associated with better outcomes per se while enabling though the shortest possible interval to a second retrieval. This evidence further strengthens the concept that DuoStim is a powerful strategy to prevent patient discontinuation and double (or more) the oocytes retrieved in a single ovarian cycle.

Regarding embryo development, couples who produced no blastocysts in their first attempt represented 33% of the cohort, which is higher than previously reported rates [12, 41]. However, prior studies were conducted in different contexts, such as day-3 embryo culture (reported risk, 4%) [41] or in younger populations (reported risk, 3%) [12]. In our study, only 13% of these patients failed to obtain blastocysts twice, as 61% of those with no embryos available during the first attempt successfully achieved blastocyst formation in the second cycle.

Overall, 45% of all patients produced more blastocysts in their second attempt. Consistent with prior evidence [41, 42], maternal age and ovarian reserve were key predictors, and our data highlight that shorter intervals between cycles also enhance blastocyst yield.

Oocyte developmental competence, defined as the blastocyst formation rate per retrieved COCs, was likewise influenced mainly by maternal age and the time between attempts. Previous studies had focused solely on maternal age without accounting for timing effects [12].

The negative influence of severe male factor infertility on fertilization and blastulation is well established [43–45]. However, our data show that even couples affected by severe male factor may achieve improved outcomes in their second attempt. This aligns with evidence suggesting that, while male factor impacts early embryo development, it does not compromise reproductive potential per euploid blastocyst transfer [43, 46]. These findings support a multicycle strategy aimed at maximizing the number of oocytes collected in a short timeframe to mitigate the impact of severe male factor.

Among patients who concluded two cycles, the chance of achieving a live birth in the second attempt was not significantly influenced by whether a live birth was achieved in the first cycle. Among the variables tested, only the number of COCs retrieved after the first ovarian stimulation and the increase in maternal age between cycles were associated with second-attempt success.

Interestingly, no modification in the ovarian stimulation protocol appeared to influence the outcomes under investigation. This observation aligns with the message of a recent manuscript intriguingly titled “Plan for the next step after a failed transfer cycle: ‘Doctor, what are we going to do differently?’” whose conclusion was essentially: “We are going to transfer a different embryo” [47]. If we extend this reasoning to an entire failed IVF attempt, the equivalent answer would be the following: “We are going to retrieve different oocytes, and the sooner we restart, the greater the likelihood of achieving improved outcomes.” In this regard, our study showed a strong correlation between the number of oocytes retrieved in the first and second cycles, a moderate correlation for blastocyst number, and only a weak correlation for blastulation rate, suggesting that while quantitative oocyte yield is consistent, qualitative competence varies substantially. Each cohort of retrieved oocytes is biologically unique and represents a new opportunity for success. Beyond maternal age, an established prognostic factor, and ovarian reserve or response to stimulation, which remain marginally modifiable, time emerges as the most crucial actionable factor, especially in time-sensitive patient populations, such as women with advanced maternal age and diminished ovarian reserve. Our study demonstrates that earlier subsequent IVF attempts are associated with improved outcomes, reinforcing the rationale for pursuing additional cycles whenever clinically reasonable [42], bearing in mind though that maternal age and ovarian reserve persist as the most powerful determinants of success.

Prolonged intervals between attempts correlate with lower success rates, both among women seeking a second ART-conceived child [48] and those resuming treatment after miscarriage [49]. Paul et al. reported that delays of ≥ 2–3 years after a first live birth were associated with progressively lower chances of a second live birth, whereas restarting within 1 year yielded better results [48]. Conversely, in women with diminished ovarian reserve, short delays (up to 180 days) from consultation to treatment did not negatively impact outcomes [50]. In our study, each 1-year increase in maternal age reduced the probability of live birth in the second cycle by ~ 11%. We also observed longer intervals between cycles following miscarriage. Previous evidence indicates that conceiving within 3–6 months after miscarriage does not increase the risk of adverse outcomes [51–53], and shorter inter-pregnancy intervals are associated with higher live birth rates in subsequent IVF cycles [49]. Consistently, our data confirm that shorter inter-cycle intervals correspond to improved outcomes, including a higher number of retrieved COCs, more blastocysts, and a greater blastocyst-to-COC ratio. Overall, these results underscore the importance of timely intervention, perhaps through a multicycle strategy, to optimize reproductive success [9–11].

The first structured application of such a strategy was the DuoStim protocol [22], adopted by 28% of the patients included in our study. Unlike conventional stimulation, DuoStim allows two oocyte retrievals within a single menstrual cycle, shortening the interval between attempts and accelerating oocyte accumulation when fresh transfer is not planned [54]. This enables patients, whether facing failure, miscarriage, or even a live birth, to invest only about 18 days to obtain an additional oocyte cohort, thereby maximizing cumulative success without prolonged waiting [55]. DuoStim can also be used as a rescue strategy after poor oocyte or blastocyst yields in a conventional stimulation cycle [55–57], offering a cost-effective option to reduce treatment discontinuation, limit further ovarian aging between attempts, and shorten time to pregnancy [58–61]. Recent studies show that DuoStim decreases time between retrievals, reduces total treatment duration, and accelerates time to live birth [55, 58, 62]. In PGT-A cycles, it might shorten the time to obtain euploid blastocysts [61, 62], ultimately supporting more efficient family planning [55].

### Limitations

We could not stratify outcomes by prognostic subgroups (e.g., POSEIDON, Bologna criteria) due to limited sample size. The mean maternal age was relatively advanced (38.7 years), and stimulation protocols (type/dose of gonadotropins, trigger method) were modified in over one-third of cases, although these variations did not appear to affect the observed trends. Moreover, these data should be interpreted bearing in mind that the women undergoing a second attempt at our center tend to be 1-year older than the general population of naïve IVF patients but are also characterized by a 0.2-point-higher basal AMH value. In other terms, these patients might be more motivated to persist with IVF because of a reasonably good ovarian reserve despite their more advanced age.

## Conclusion

In second IVF attempts, the chances of retrieving more COCs, obtaining more blastocysts, and achieving ≥ 1 live birth are independent of the first attempt outcomes. When clinically feasible, couples should be reassured that a previous negative result does not compromise future success. Furthermore, shorter intervals between cycles are associated with improved outcomes, emphasizing the need for timely intervention, especially as maternal age and ovarian reserve remain the most important predictors of success in second attempts. Adopting a multicycle counseling approach from the very first consultation is therefore essential to prevent premature discontinuation after a first IVF failure.

## Supplementary Information

Below is the link to the electronic supplementary material.

ESM 1 (33.2 KB PDF)

ESM 2 (136 KB PDF)

## Data Availability

The data supporting this article are available within the article and in its online supplementary material.
