# Supplementary material for: Another round, another chance: oocyte developmental competence and outcomes in second IVF attempts—the earlier, the better
Source: J Assist Reprod Genet. 2026 Jul 3;43(8):2443–55. doi: 10.1007/s10815-026-03961-8 (PMC13597993; doi:10.1007/s10815-026-03961-8)
Supplement: Supplementary file 1 [file 10815_2026_3961_MOESM1_ESM.pdf]

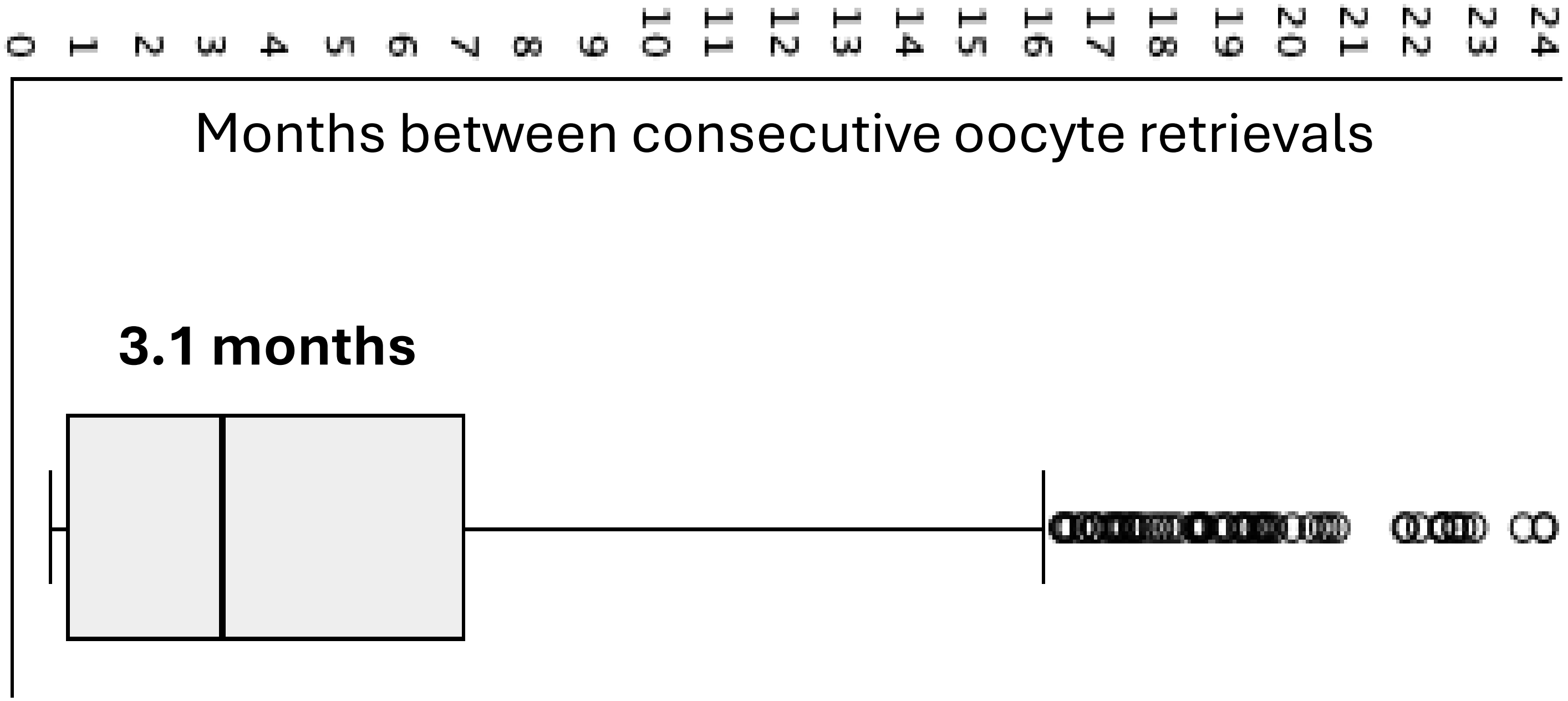

0
1
2
3
4
5
6
7
8
9
10
11
12
13
14
15
16
17
18
19
20
21
22
23
24
Months between consecutive oocyte retrievals
3.1 months
