# Supplementary material for: Another round, another chance: oocyte developmental competence and outcomes in second IVF attempts—the earlier, the better
Source: J Assist Reprod Genet. 2026 Jul 3;43(8):2443–55. doi: 10.1007/s10815-026-03961-8 (PMC13597993; doi:10.1007/s10815-026-03961-8)

A

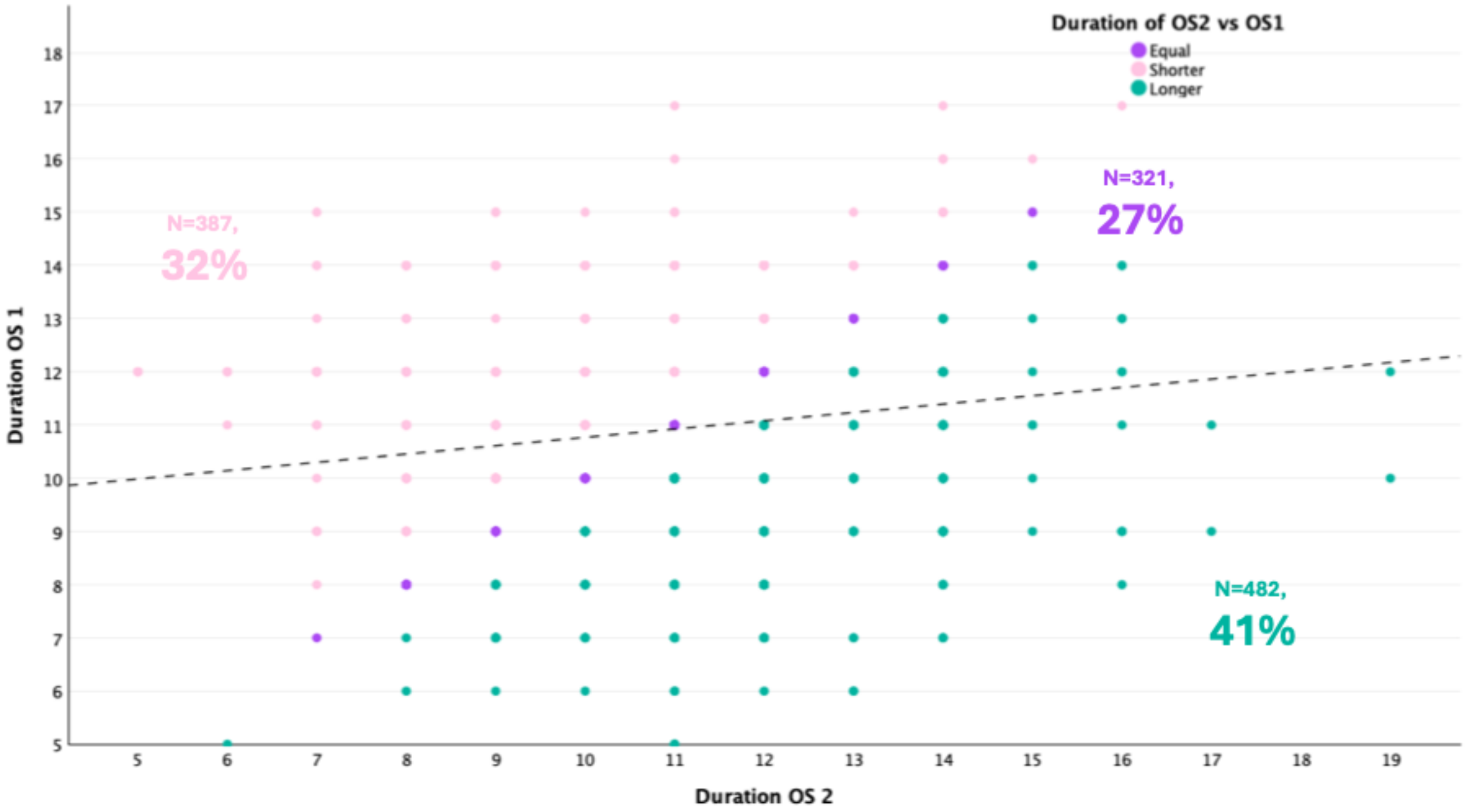

B

		FSH starting dose OS 2		
		150 IU	225 IU	300 IU
FSH starting dose OS 1	150 IU	N=101<br>9%	N=33<br>3%	N=47<br>4%
	225 IU	N=33<br>3%	N=60<br>5%	N=129<br>11%
	300 IU	N=95<br>8%	N=77<br>7%	N=615<br>52%

C

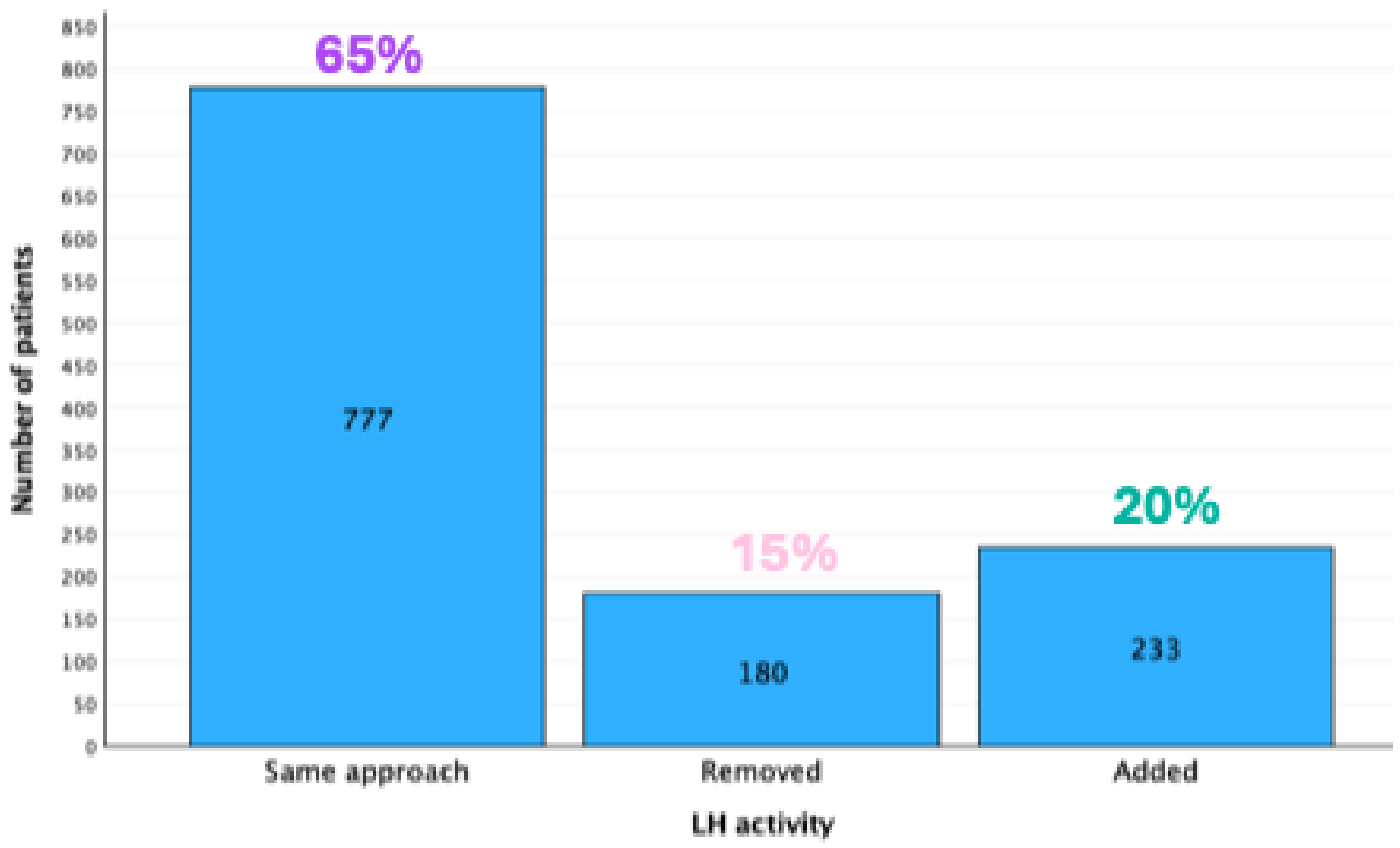

D

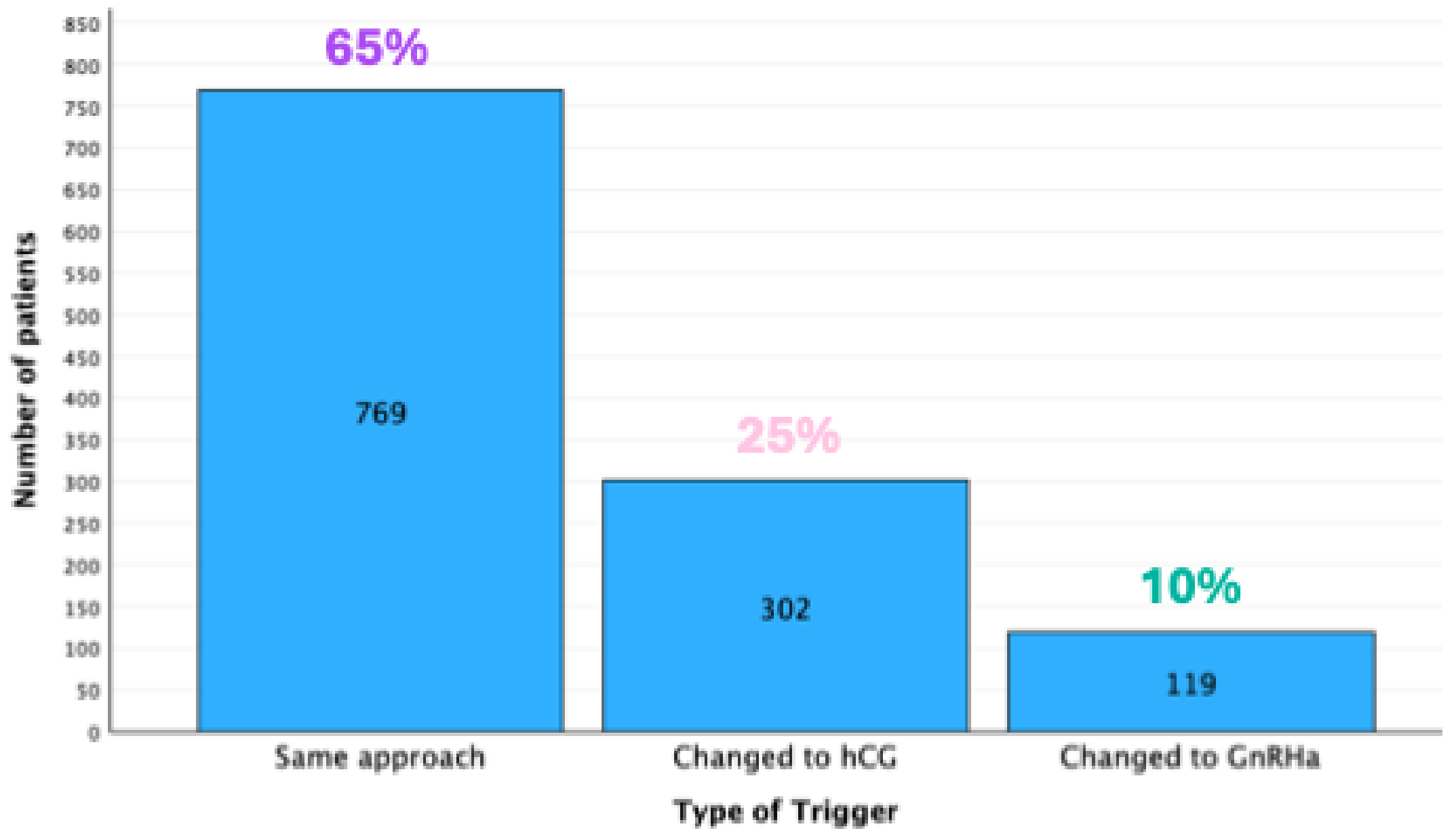

Supplement: Supplementary file 2 [file 10815_2026_3961_MOESM2_ESM.pdf]
